# Low-dose tamoxifen ameliorates ovariectomy-induced metabolic and immune dysfunction

**DOI:** 10.1007/s11357-025-02086-7

**Published:** 2026-02-11

**Authors:** Heather E. McClurg, Amanda Ferraro, Gina Pham, Itzel Morales-Hernandez, Estefania Valencia-Rincon, Kevin D. Pham, Willard M. Freeman, Jacquelyn A. Gorman, Marisol Castillo-Castrejon

**Affiliations:** 1 Department of Pathology, The University of Oklahoma College of Medicine, Biomedical Research Center, Room 458, 975 NE 10 Street, Oklahoma City, OK 73104 USA; 2 Section of Reproductive Endocrinology and Infertility, Department of Obstetrics and Gynecology, The University of Oklahoma College of Medicine, Oklahoma City, OK USA; 3 Genes and Human Disease Research Program, Oklahoma Medical Research Foundation, Oklahoma City, OK USA; 4 Oklahoma City Veterans Affairs Medical Center, Oklahoma City, OK USA; 5 Arthritis & Clinical Immunology Research Program, Oklahoma Medical Research Foundation, Oklahoma City, OK USA; 6 Center for Geroscience and Healthy Brain Aging, University of Oklahoma Health Sciences Center, Oklahoma City, OK 73104 USA

**Keywords:** Estrogen, Menopause, B cells, Adipose tissue, Inflammation

## Abstract

The decline in estrogen following menopause is a major driver of metabolic and immune dysfunction in aging females. While hormone replacement therapy improves many of these outcomes, its clinical use remains limited due to concerns regarding estrogen-sensitive malignancies. Tissue-selective estrogen complexes (TSECs), which combine estrogens with selective estrogen receptor modulators (SERMs) such as tamoxifen (TAM), represent a promising strategy to preserve the metabolic and immunological benefits of estrogen while reducing oncogenic risk. However, the systemic effects of TAM under conditions of estrogen deficiency remain incompletely defined. In this study, we investigated the metabolic and immunomodulatory actions of low-dose TAM, alone or in combination with 17β-estradiol (E2), in adult ovariectomized (OVX) female mice. OVX resulted in increased adiposity, hepatic steatosis, glucose intolerance, insulin resistance, immunoglobulin G3 (IgG3) concentration, and systemic inflammation, along with decreased immunoglobulin G1 (IgG1) concentration. E2, TAM, and E2 + TAM each attenuated OVX-induced adipose expansion, adipocyte hypertrophy, and proinflammatory cytokine production. TAM improved insulin sensitivity but did not fully restore glucose tolerance. Transcriptomic analysis of visceral adipose tissue-resident B cells revealed that E2 and E2 + TAM modulate overlapping yet distinct immune-regulatory networks, including suppression of pro-inflammatory signaling, regulation of immune checkpoints, and genes linked to adipose homeostasis. Low-dose TAM emerges as a modulator of estrogen receptor signaling that attenuates multiple features of OVX-induced metabolic and inflammatory dysfunction, though its limited effect on glycemic control highlights the need for tissue-specific evaluation of SERM-based interventions. These findings provide mechanistic insight into endocrine–immune–metabolic interactions and inform strategies to reduce aging-related disease risk in postmenopausal women.

**Graphical Abstract:**

Created in BioRender (https://BioRender.com/7ouhlqq), Castillo M. (2025)

**Figure:**
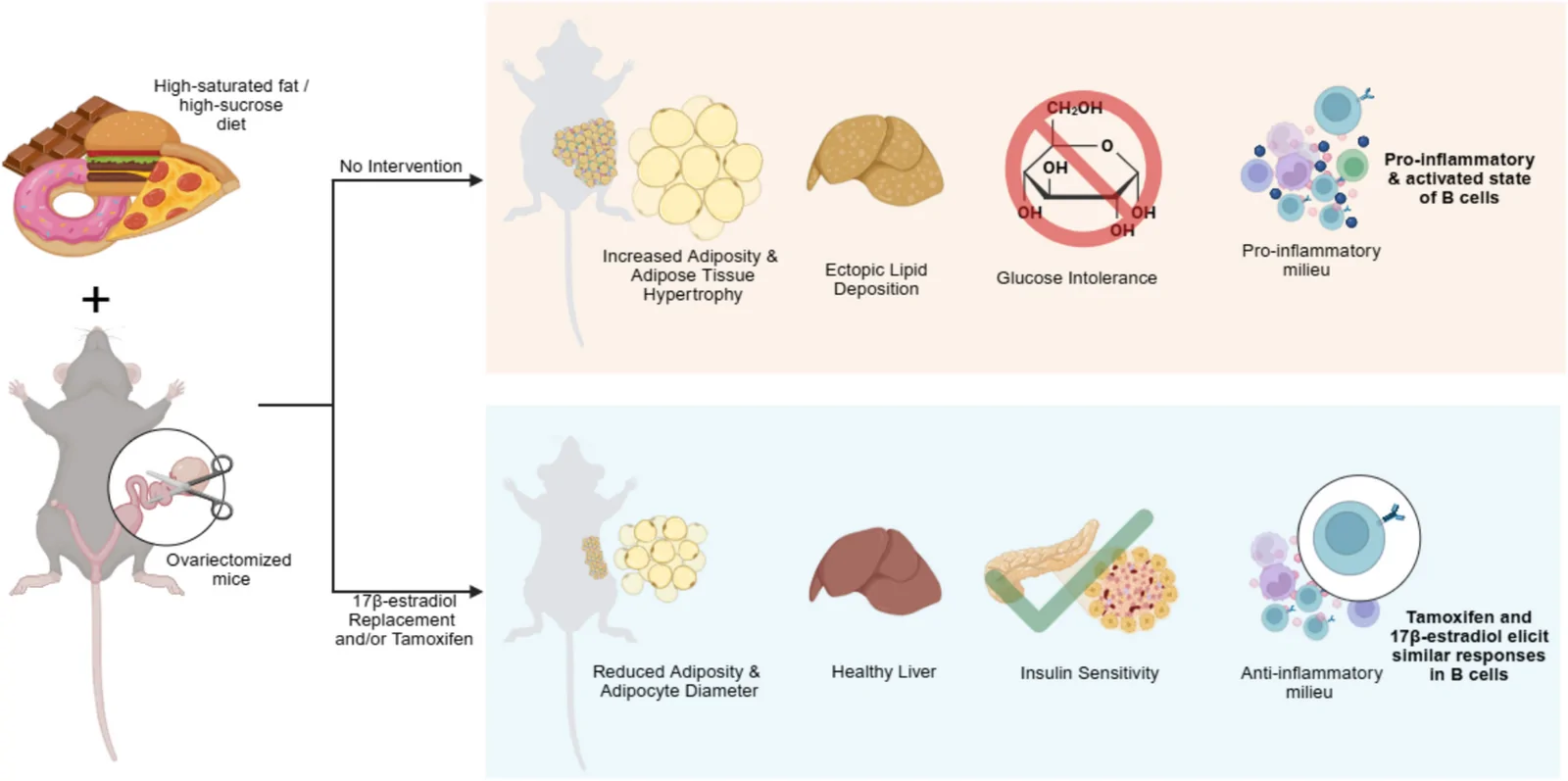

**Supplementary Information:**

The online version contains supplementary material available at https://doi.org/10.1007/s11357-025-02086-7.

## Introduction

Menopause, defined as the permanent cessation of menstruation and clinically recognized as 12 consecutive months of amenorrhea, marks the end of a woman’s reproductive years. It typically occurs between the ages of 45 and 55, with a median age of 51 in the United States [1]. With increasing life expectancy, approximately one-third of a woman’s life is now spent in a postmenopausal state [2]. The menopausal transition is characterized by the loss of ovarian follicular function and a marked decline in sex steroid hormones, particularly 17β-estradiol (E2) and progesterone [1]. This hormonal shift is associated with increased adiposity, reduced metabolic flexibility, and a higher risk of age-related diseases including obesity [3], type 2 diabetes mellitus (T2DM) [4], cardiovascular disease (CVD) [5], and hormone-sensitive cancers such as breast cancer [6, 7].

Postmenopausal estrogen deficiency is increasingly recognized as a key contributor to metabolic dysfunction in aging women. The decline in circulating E2 promotes visceral fat accumulation, decreases lean mass, and contributes to central obesity [8]. Estrogen plays a central role in regulating whole-body energy homeostasis through its actions on estrogen receptors (ERα and ERβ), which are broadly expressed across tissues. These receptors mediate the effects of estrogen on food intake, energy expenditure [9], glucose and lipid metabolism [10], adipocyte biology [11], and immune regulation [12, 13]. The wide tissue distribution of ERs—including in the brain, liver [14], adipose tissue (AT) [15], and the immune system [13] highlights the systemic impact of E2 beyond reproductive function.

Menopause also leads to pronounced changes in immune function. Estrogen signaling modulates both innate and adaptive immunity, and its decline contributes to immunological aging [16, 17]. In both obesity and aging, immune cell infiltration into adipose tissue (AT) is a hallmark of metabolic dysfunction, promoting a proinflammatory microenvironment [18–20]. B cells have emerged as key regulators of metabolic health through their influence on glucose metabolism, weight gain, and their interaction with AT-resident T cells and macrophages [21]. Increased AT B cell infiltration has been observed in obesity [22, 23], aging [24], and menopause [25]. Moreover, adipose tissue alters B cell phenotype by increasing the frequency of proinflammatory age-associated B cells [26] and modifying B cell metabolic function [27]. Given that B cells express the highest levels of ERs among immune cells [28], they are particularly sensitive to hormonal fluctuations. Changes in peripheral immune cells after menopause have been attributed to E2 loss; some of these changes are linked to an increased risk of cardiovascular and autoimmune diseases, and T2D [29].

Hormone replacement therapy (HRT), composed of estrogen alone or combined with progesterone [30], has been shown to alleviate vasomotor symptoms [31], improve body composition [32, 33], and potentially reverse menopause-associated immune aging by restoring B cell numbers, class-switched memory B cells, and T cell responsiveness [34, 35]. However, concerns about the increased risk of estrogen-sensitive cancers such as endometrial and breast cancer limit universal HRT use [36]. Tissue-selective estrogen complexes (TSECs) offer an alternative approach to traditional HRT. TSECs combine estrogens with selective estrogen receptor modulators (SERMs) such as tamoxifen (TAM) to maximize therapeutic benefit while minimizing estrogenic stimulation of reproductive tissues [37]. SERMs are nonsteroidal compounds that exhibit tissue-specific ER agonist or antagonist activity depending on the target organ [38, 39]. TAM, widely used in the treatment and prevention of ER-positive breast cancer, functions as an antagonist in breast tissue and as an agonist in other tissues, such as bone and endometrium [40, 41]. While TAM’s role in oncology is well established [42], its effects on systemic metabolism and immune regulation, particularly with estrogen deficiency or replacement, remain poorly understood. This understanding is further limited when TAM is utilized in conjunction with estrogen, as in a TSEC.

Given the limitations of conventional HRT and the variability in individual response, further investigation into the estrogen-regulated pathways driving metabolic and immune dysfunction in menopause is warranted. In this study, we examined the effects of ovariectomy-induced estrogen deficiency and ER modulation by low-dose TAM on body composition, metabolic function, and adipose tissue B-cell molecular phenotype in adult female mice. We assessed whether TAM could prevent OVX-induced metabolic dysfunction and evaluated its immunomodulatory effects on B cells isolated from visceral adipose tissue.

We demonstrated that OVX mice developed hallmark features of metabolic dysfunction, including increased body weight and fat mass, adipocyte hypertrophy, ectopic lipid deposition, systemic inflammation, glucose intolerance, and insulin resistance. In contrast, low-dose TAM, both alone and in combination with E2, mitigated many of these phenotypes, producing effects similar to E2 replacement. Notably, although glucose tolerance remained impaired in TAM-treated mice, insulin sensitivity was restored to normal. Transcriptomic profiling of adipose tissue B cells revealed overlapping gene expression changes between E2 and TAM treatment groups, suggesting that TAM mimics key immunometabolic actions of E2. These findings support the role of low-dose TAM as a modulator of metabolic and immune function during estrogen deficiency.

## Materials and methods

### Experimental animals

Female C57BL/6 J mice (Strain #000664) were obtained from Jackson Laboratories at 6 weeks of age. Mice were housed five per cage under standard conditions and acclimated for 1 week before being transitioned to an ad libitum high-fat/high-sucrose (HF/HS) diet (40% kcal from fat; 292.5 g sucrose per 3902 kcal; Research Diets D15031601). To promote diet-induced weight gain, animals were housed on warming blankets under thermoneutral conditions, as described [43]. At 32 weeks of age, mice underwent bilateral ovariectomy via dorsal incision under isoflurane anesthesia (2.5%). The ovariectomized (OVX) mouse model is widely used to study postmenopausal physiology, as it closely mimics the hormonal environment of estrogen deficiency in women [44]. Following a 2-week postoperative recovery period, mice were randomized into four treatment groups with similar average body weights: (1) OVX (no treatment), (2) OVX + E2 (+ E2, 17β-estradiol 1 µM in drinking water), (3) OVX + tamoxifen (+ TAM; 5 mg 60-day release pellet implanted subcutaneously (low-dose tamoxifen); Innovative Research of America, Cat. No. SE-361), and (4) OVX + E2 + TAM (+ E2 + TAM). The tamoxifen dosage was based on allometric scaling from human low-dose trials [45]. Treatments were administered for 10 weeks. At the end of the study period, mice were fasted for 12 h and euthanized by CO₂ asphyxiation followed by cervical dislocation. Blood was collected via cardiac puncture, and serum was isolated and stored at − 80 °C for downstream analyses. Eight to nine mice per group completed the study. Mice were then perfused with ice-cold 1 × PBS, and tissues were excised, weighed, flash-frozen in liquid nitrogen, and stored at − 80 °C. All procedures were approved by the University of Oklahoma Institutional Animal Care and Use Committee (IACUC Protocol #23–011-SEH).

### Body composition assessment

Whole-body composition was assessed via magnetic resonance imaging using the EchoMRI 2020 Body Composition Analyzer (Echo Medical Systems, Houston, TX). Live mice were scanned at two timepoints: prior to ovariectomy and again after 10 weeks of treatment. Each scan was performed with a primary accumulation time of 1 min.

### Intraperitoneal glucose tolerance test (GTT) and insulin tolerance test (ITT)

To evaluate glucose metabolism and insulin sensitivity, mice were fasted for 4 h before undergoing intraperitoneal glucose tolerance test (GTT) and 5 h before insulin tolerance test (ITT), with 1 week separating the tests. Mice received an intraperitoneal injection of either glucose (2 g/kg) for the IPGTT or insulin (0.75 mU/g; Novolin-R, 100 U/mL, Novo Nordisk) for the ITT. Blood glucose was measured from tail vein blood at baseline and 15, 30, 60, 90, and 120 min post-injection using a Contour Next EZ glucometer (Ascensia). Glucose tolerance and insulin sensitivity were quantified by calculating the area under the curve (AUC) for each test. Fasting insulin levels were measured from blood collected at euthanasia using a mouse-specific Ultrasensitive Insulin ELISA (Alpco, Salem, NH, USA, Cat. No. 80-INSMSU-E01).

### Adiposity and ectopic lipid deposition histological analysis

Following 10 weeks of treatment, mice were euthanized, and subcutaneous (mammary gland) and visceral (mesenteric and perigonadal) white adipose tissue (WAT), as well as liver tissue, were harvested. Tissues were fixed in 10% neutral buffered formalin for 24 h, processed, and embedded in paraffin. Sections (4 µm) were stained with hematoxylin and eosin (H&E) and scanned using a Zeiss AxioScan.Z1 slide scanner.

Adipocyte cellularity was assessed by analyzing five to eight randomly selected regions (1664 × 1135 µm) per sample in subcutaneous and mesenteric WAT. Adipocyte diameter and cell number were quantified using ImageJ (NIH, Bethesda, MD).

Ectopic hepatic lipid accumulation was analyzed by dividing each liver section into quadrants along its longest and shortest diameters, then selecting two regions per quadrant plus one central region (nine total regions per liver). Lipid droplet area was measured using the “Analyze Particles” tool in ImageJ (NIH, Bethesda, MD). Prior to analysis, all images were 8-bit converted, background noise was removed, and binary thresholds were set. A macro was developed to exclude non-lipid structures (e.g., blood vessels), and post hoc outlier analysis was conducted to ensure specificity.

### Quantification of insulin-secreting pancreatic islets

Pancreatic tissue was fixed in 10% neutral buffered formalin, embedded in paraffin, and sectioned at 4 µm for immunohistochemistry (IHC). Slides were deparaffinized in xylene, rehydrated through graded ethanol, and subjected to heat-induced antigen retrieval using Tris/EDTA buffer. Sections were blocked for peroxidase activity and nonspecific binding, followed by incubation with a primary anti-insulin rabbit monoclonal antibody (Abcam, Cat. No. ab181547; 1:100,000 dilution). Following primary antibody incubation, slides were treated with horseradish peroxidase polymer and developed with 3,3′-diaminobenzidine (ImmPRESS HRP Horse Anti-Rabbit IgG Polymer Kit, Vector Laboratories, Cat. No. MP-7801). Counterstaining was performed using Mayer’s hematoxylin (Sigma-Aldrich, Cat. No. MHS1-100ML), and slides were mounted with toluene-based mounting medium (Permount, Fisher Scientific, Cat. No. SP15-100). Slides were scanned using a Zeiss AxioScan.Z1. Image analysis was conducted in ImageJ (NIH, Bethesda, MD) using Weka segmentation followed by binary thresholding and particle analysis to determine islet number, size, and total insulin-positive area per image.

### Visceral adipose tissue stromal vascular fraction isolation and B cell enrichment

Perigonadal visceral adipose tissue (VAT) was collected and rinsed in adipose tissue (AT) wash buffer (HBSS with Ca^2^⁺/Mg^2^⁺ + 3% BSA + 800 µM ZnCl₂). Tissues were minced and digested in warmed digestion buffer (AT wash buffer with 0.8 mg/10 mL collagenase) at 37 °C with gentle agitation (100 RPM) for 30 min. The digested suspension was filtered through a 70-µm mesh, and red blood cells were lysed using Red Blood Cell Lysing Buffer Hybri-Max (Sigma-Aldrich, Cat. No. R7757-100ML). Cell yield was quantified using a Bio-Rad TC20 Automated Cell Counter. B cells were then isolated by negative magnetic selection using the Pan B Cell Isolation Kit II (Miltenyi Biotec, Cat. No. 130–104-443), following manufacturer instructions. Briefly, cells were incubated in Pan B buffer with FcR Blocking Reagent and a biotin-antibody cocktail at 4 °C, followed by labeling with anti-biotin microbeads. Magnetic separation was performed using the autoMACS Pro Separator (Deplete program, Miltenyi Biotec). Isolated B cells were lysed in RLT buffer (QIAGEN, Cat. No. 79216) and stored at − 80 °C for downstream transcriptomic analyses.

### RNA sequencing and bioinformatics

RNA was isolated from perigonadal fat-derived B cells using the RNeasy Micro Kit (QIAGEN, Cat. No. 74004) according to the manufacturer’s instructions. RNA integrity was assessed using an Agilent TapeStation 4250 to ensure adequate quality prior to RNA sequencing (RNA-Seq) library preparation. Directional RNA-Seq libraries were generated using the NEBNext Ultra II Directional RNA Library Prep Kit (New England Biolabs, Ipswich, MA; NEB Cat. No. E7760), as previously described [46]. Polyadenylated mRNA was selectively enriched using the NEBNext Poly(A) mRNA Magnetic Isolation Module (Cat. No. NEB E7490). Following mRNA capture, the RNA was eluted from oligo-dT beads and fragmented by incubation in a first-strand synthesis reaction buffer with Random Primer Mix (2 ×) at 94 °C for 15 min. First- and second-strand cDNA synthesis was performed according to the manufacturer’s protocol. Purified double-stranded cDNA was eluted in 50 µL 0.1 × TE buffer and subjected to end repair. NEBNext adaptors were ligated to the cDNA and then purified using 0.9 × SPRISelect beads (Cat. Beckman Coulter, No. B23318). Libraries were amplified with 15 cycles of PCR using NEBNext Ultra II Q5 Master Mix (provided) and unique index primer sets from the NEBNext Multiplex Oligos for Illumina (New England Biolabs, Cat. No. E6609L). Amplified libraries were purified again using SPRISelect beads, sized with the HSD1000 ScreenTape (Agilent Technologies, Cat. No.5067–5584), and quantified using the Qubit 1 × dsDNA HS Assay kit (Thermo Fisher, Cat. No,Q33230). Libraries were pooled at 4 nM concentration and sequenced on an Illumina NovaSeq X system (PE150). Sequencing data are available in FASTQ format for download from the NCBI Gene Expression Omnibus (GEO; GSE313405).

After sequencing, reads were aligned against the mm39 transcriptome and genome in StrandNGS software (v4.1; Strand Life Sciences). Parameters for alignment and filtering included: adapter trimming, fixed 2-bp trim from 5′ and 3′ ends, a maximum number of one novel splice allowed per read, a minimum of 90% identity with the reference sequence, a maximum 5% gap, and trimming of 3′ ends with *Q* < 30. Alignment was performed directionally with Read 1 aligned in reverse and Read 2 in the forward orientation, as previously described [47]. Binary alignment map (BAM) files were exported from StrandNGS software for further analysis. The Rsubread package was used to quantify the read counts using the featureCount command. Normalization and differential expression calling was then performed using DESeq2 as described previously [48]. Transcripts with an average read count value > 5 in at least 100% of the samples in at least one group were considered expressed at a level sufficient for quantitation per tissue and those transcripts below this level were considered not detected/not expressed and excluded, as these low levels of reads are close to background and are highly variable. For statistical analysis of differential expression, an ANOVA between groups with Benjamini–Hochberg multiple testing correction (BHMTC) was performed followed by pairwise SNK post-hoc testing. For those transcripts meeting this statistical criterion, a fold change >|1.25| cutoff was used to eliminate those genes which were statistically significant but unlikely to be biologically significant and orthogonally confirmable because of their very small magnitude of change. Visualizations of hierarchical clustering and principal component analyses (PCAs) were performed in Strand, and IPA.

### Systemic inflammation parameters assessment

Systemic inflammation was assessed in serum using a custom-made Milliplex MAP Mouse High-Sensitivity T-Cell Magnetic Bead Panel (Millipore Sigma, Darmstadt, Germany, Cat. No. MHSTCMAG-70 K), following the manufacturer’s guidelines. Briefly, pre-washed magnetic beads were incubated overnight at 4 °C with agitation (800 RPM). After incubation, detection antibodies were added, followed by streptavidin–phycoerythrin. After final incubation, bead counts were determined using a BioPlex 200 Multi-Array System (Bio-Rad Laboratories, Hercules, CA). Analyte concentrations were calculated based on median fluorescence intensity, and a five-parameter logistic curve was used for quantification. Minimum detection concentrations (pg/mL) were as follows: Interleukin-6 (IL-6), 0.54; Interleukin-17A (IL-17A), 0.488; Tumor necrosis factor alpha (TNF-α), 0.41; Interleukin-12 (IL-12p70), 1.29; Interleukin-4 (IL-4), 0.06; Interferon gamma (IFN-γ), 0.15.

### Estradiol levels

Serum estradiol (E2) concentrations were measured using the competitive 17β-Estradiol Serum ELISA kit (Arbor Assays, Ann Arbor, MI, Cat. No. KB30-H1) following the manufacturer’s protocol. A standard curve was generated using estradiol standards (3.75–120 pg/mL), and serum samples were diluted 1:20 before being added to a donkey anti-sheep IgG coated 96-well plate. An estradiol-peroxidase conjugate was added, and after a 2-h incubation, the plate was washed and treated with TMB substrate. After a final 30-min incubation, the reaction was stopped with 1 M HCl, and absorbance was measured at 450 nm using a Synergy HTX multi-mode plate reader (Agilent Technologies, Santa Clara, CA). Estradiol concentrations were determined using a four-parameter logistic curve.

### Quantification of immunoglobulin G subclasses

Plasma immunoglobulin G (IgG), immunoglobulin G1 (IgG1), and immunoglobulin G3 (IgG3) concentrations were measured by ELISA. 96-well plates were coated with 10 µg/mL goat anti-mouse IgG + IgM (Jackson ImmunoResearch, 115–005-044, Cat. No. NC1444091) diluted in carbonate binding buffer and incubated overnight at 4 °C. Plates were washed with PBS containing 0.05% Tween-20 (PBST) and blocked with 1% BSA in PBST for 2 h at room temperature. Plasma samples were diluted 1:100, 000 for IgG and 1:10,000 in PBST for IgG1 and IgG3. Standards (mouse IgG1-UNLB, SouthernBiotech, Cat. No. 0102–01; mouse IgG3-UNLB, SouthernBiotech, Cat. No. 0105–01) were prepared at 0.0625 µg/mL or 0.25 µg/mL, respectively, in PBST and serially diluted. IgG standards (mouse IgG-UNLB, SouthernBiotech, Cat. No. 0107–01) were prepared at 0.2 µg/mL in PBST and serially diluted. A total of 100 µL of standards or diluted plasma was added per well and incubated overnight at 4 °C. Following washing, HRP-conjugated goat anti-mouse IgG, IgG1, or IgG3 (SouthernBiotech, Cat. No. 1030–05, 1070–05, and 1100–05, respectively) was applied for 1 h at room temperature in the dark. ABTS substrate (SeraCare Life Sciences Inc 5120–0046, Cat. No. 50-674-17) was added, and color development was monitored for 5 min before terminating the reaction with excess 10% SDS. Absorbance was measured at 405 nm using a Synergy HTX multi-mode plate reader (Agilent Technologies, Santa Clara, CA). IgG and subclass concentrations were calculated using a five-parameter logistic standard curve.

### Statistics and analysis

Data were analyzed using one-way analysis of variance (ANOVA) to compare the means of all groups. The assumptions of normality and homogeneity of variance were verified before performing ANOVA. When a significant difference was detected, Sidak’s multiple comparison test or Fisher’s LSD test was applied to compare individual group means. All statistical analyses were performed using GraphPad Prism software (Boston MA, version 10.3.1). Statistical significance was set at *p* < 0.05.

## Results

### Tamoxifen ameliorates OVX-induced body weight gain and adiposity similar to 17β-estradiol

**Fig. 1:**
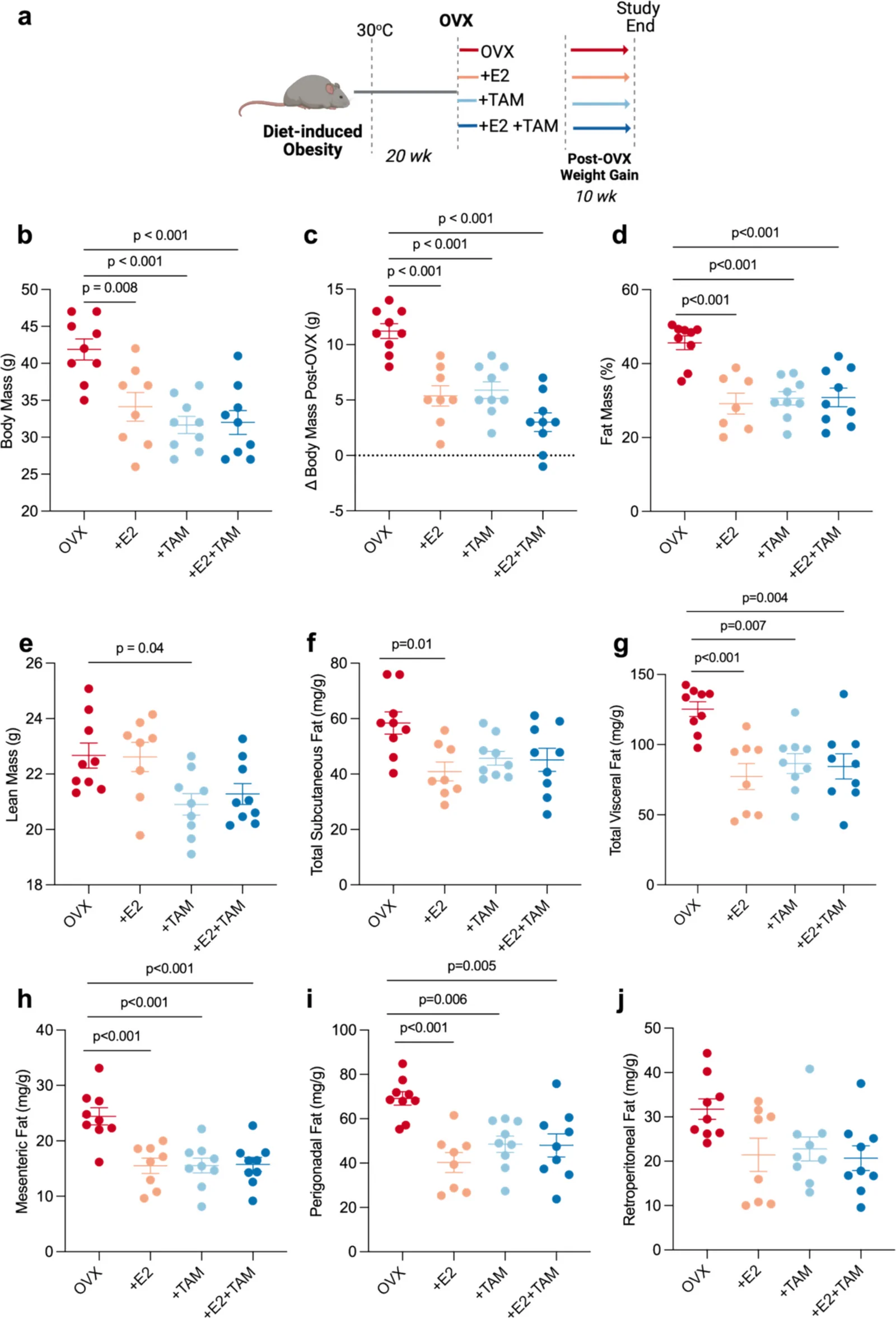
Tamoxifen and estradiol treatment modulate OVX-induced changes in body composition. **a** Experimental timeline: Female C57BL/6 J mice were placed on a high-fat/high-sucrose diet (HF/HS) for 20 weeks to induce obesity, then randomized into four treatment groups following ovariectomy and treated for 10 weeks. **b** Final total body mass at study endpoint; **c** total change in body mass from ovariectomy to study conclusion; **d** final body fat percentage; **e** final lean mass (g); **f** subcutaneous fat mass (mammary depot) normalized to body mass (mg/g); **g** combined visceral fat mass (mesenteric, perigonadal, and retroperitoneal depots) normalized to body mass (mg/g); **h** mesenteric fat mass normalized to body mass (mg/g); **i** perigonadal fat mass normalized to body mass (mg/g); and **j** retroperitoneal fat mass normalized to body mass (mg/g); one-way ANOVA, Sidak’s multiple comparison test; data are presented as mean ± SEM. OVX, *n* = 9; + E2, *n* = 8; + TAM, *n* = 9; + E2 + TAM, *n* = 9; E2 = 1 µM in drinking water; TAM = 5 mg, 60-day release pellet

We assessed the effects of 17β-estradiol (+ E2), low-dose tamoxifen (+ TAM), and their combination (+ E2 + TAM) on body composition in ovariectomized (OVX) female mice (Fig. 1a). As anticipated, the OVX mice showed significant increases in body weight and fat deposition compared to the + E2-treated mice. The OVX mice gained an average of 11.2 g, 68% of which was fat mass, whereas the + E2 mice gained only 5.4 g, with fat accounting for 6% of the total gain. Intriguingly, the + TAM mice displayed weight gain of 5.9 g, with 35% attributable to fat, while the + E2 + TAM group gained 3.0 g without measurable fat accumulation (Fig. 1b–d). We also examined body fat redistribution by dissecting visceral (mesenteric and perigonadal) and subcutaneous white adipose tissues. OVX mice exhibited a 1.5-fold increase in visceral adipose tissue compared to all other groups (Fig. 1g) and 1.4-fold more subcutaneous fat compared to the + E2 group (Fig. 1f). Similarly, OVX mice accumulated more mesenteric and perigonadal AT compared to the +E2 replacement, + TAM treatment alone, or their combination. No significant differences were observed in retroperitoneal fat across the study groups (Fig. 1j). These results suggest that OVX induces pronounced changes in body composition, which are ameliorated by TAM treatment, mimicking the effects of E2. Moreover, combining TAM with E2 did not produce a synergistic effect. As expected, OVX led to a reduction in uterine mass, whereas E2 replacement induced uterine hyperplasia. In line with the anti-estrogenic effects of TAM on uterine tissue, the combination of + E2 + TAM substantially suppressed E2-induced uterine hyperplasia and prevented the OVX-induced decrease in uterine mass (Suppl Fig. 1a). Serum estradiol concentrations confirmed elevated levels in the E2 replacement groups (Suppl Fig. 1b).

**Fig. 2:**
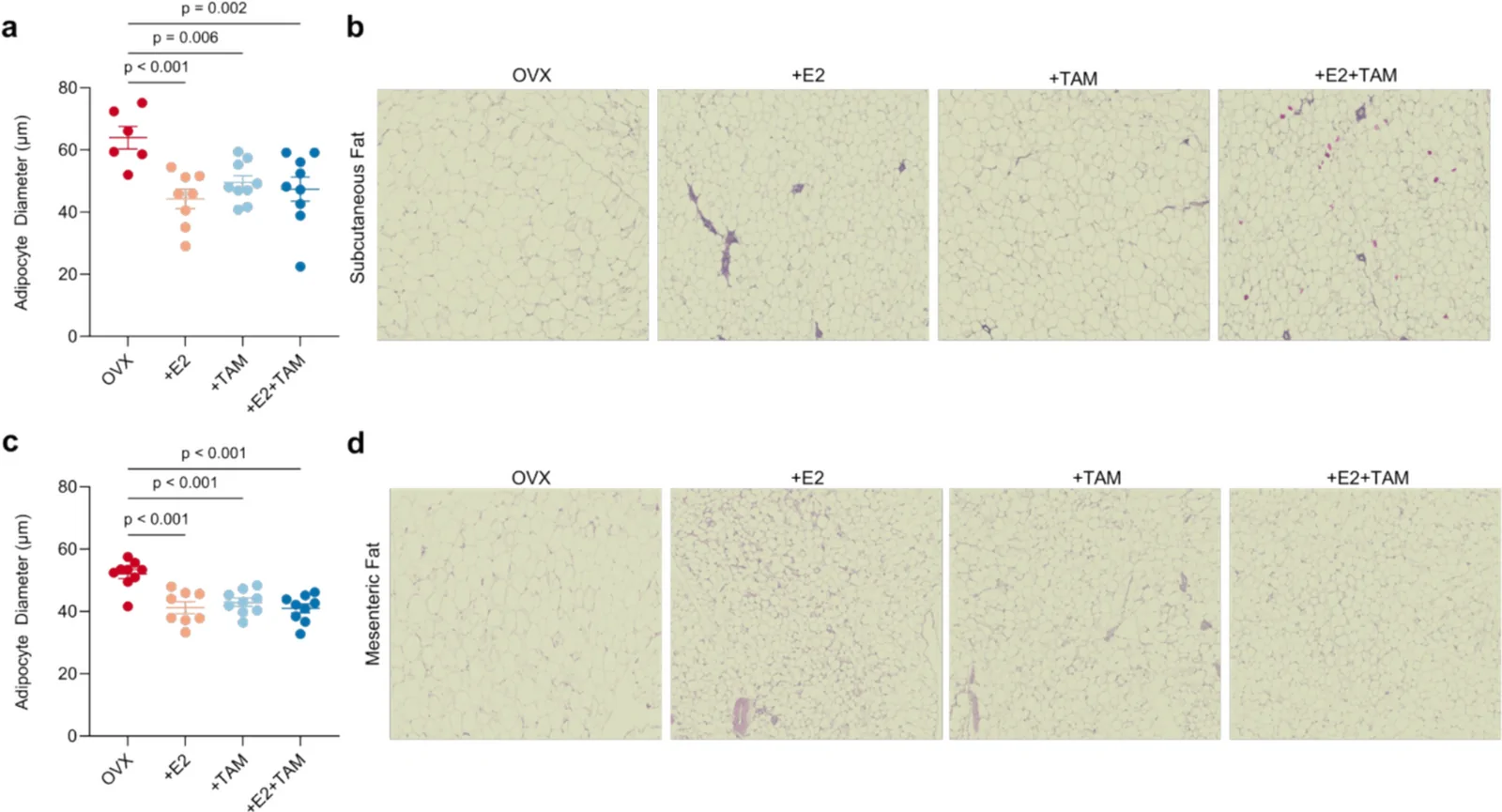
Ovariectomy increases adipocyte size, which is ameliorated by tamoxifen and estradiol treatment. **a** Average adipocyte diameter (µm) in subcutaneous adipose tissue; each point represents an individual animal. **b** Representative hematoxylin and eosin (H&E)-stained images of subcutaneous adipose tissues at × 20 magnification showing adipocyte morphology in the OVX (left), + E2 (center left), + TAM (center right), and + E2 + TAM (right) groups. **c** Average adipocyte diameter (µm) in mesenteric visceral adipose tissue; each point represents an individual animal. **d** Representative H&E-stained images of mesenteric fat at × 20 magnification for the OVX (left), + E2 (center left), + TAM (center right), and + E2 + TAM (right) groups. One-way ANOVA, Sidak’s multiple comparison test; data are presented as mean ± SEM. OVX, *n* = 9; + E2, *n* = 8; + TAM, *n* = 9; + E2 + TAM, *n* = 9

Histological analysis of adipose tissue cellularity revealed that OVX mice had significantly larger adipocytes in both visceral and subcutaneous depots compared to all treatment groups. Notably, subcutaneous adipocytes exhibited the largest diameter compared to the mesenteric depot, highlighting depot-specific responses to OVX (Fig. 2). TAM treatment, both alone and in combination with E2, reduced adipocyte diameters by 26% in the subcutaneous depot (Fig. 2a) and 21% in the mesenteric depot (Fig. 2c), mirroring the effects of E2 replacement.

### Tamoxifen Treatment Shows Hepatic Lipid Deposition Similar to OVX

**Fig. 3:**
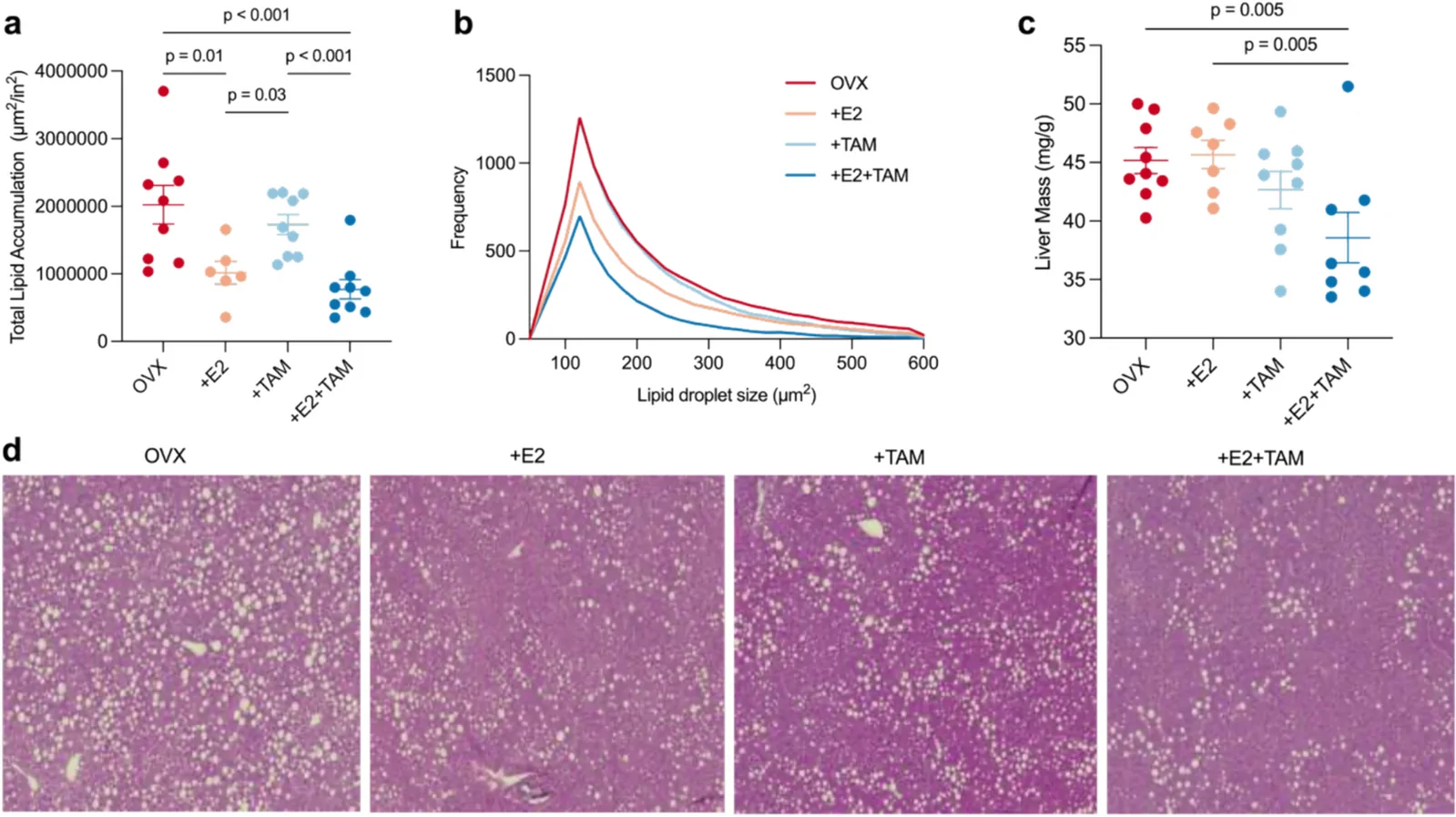
Tamoxifen and OVX-induced ectopic liver lipid deposition is mitigated by estradiol. **a** Quantification of average liver lipid droplet area per tissue section (µm^2^/in^2^); each data point represents an individual animal. **b** Distribution histogram of individual lipid droplet sizes (µm^2^) by total count. **c** Liver mass normalized to body mass (mg/g) at study endpoint. **d** Representative H&E-stained liver sections at 20 × magnification showing lipid droplets (off-white) within the liver tissue (pink) across treatment groups: OVX (left), + E2 (center left), + TAM (center right), and + E2 + TAM (right). One-way ANOVA, Sidak’s multiple comparison test; data are presented as mean ± SEM. OVX, *n* = 9; + E2, *n* = 8; + TAM, *n* = 9; + E2 + TAM, *n* = 9

We assessed liver ectopic hepatic lipid deposition using histological analysis. The OVX mice exhibited nearly double the hepatic lipid content compared to the + E2 mice (Fig. 3a). Mice receiving TAM showed 1.7 times more hepatic lipid deposition than the + E2 mice, a value comparable to the OVX mice. E2 replacement similarly decreases lipid deposition independent of TAM treatment. Furthermore, the OVX and + TAM groups exhibited higher frequencies of lipid droplets compared to the + E2 and + E2 + TAM groups (Fig. 3b), although lipid droplet size distributions were similar across all groups. Liver mass remained unaffected among most groups, except for

### Tamoxifen Treatment Impairs Glucose Tolerance but Not Insulin Sensitivity

**Fig. 4:**
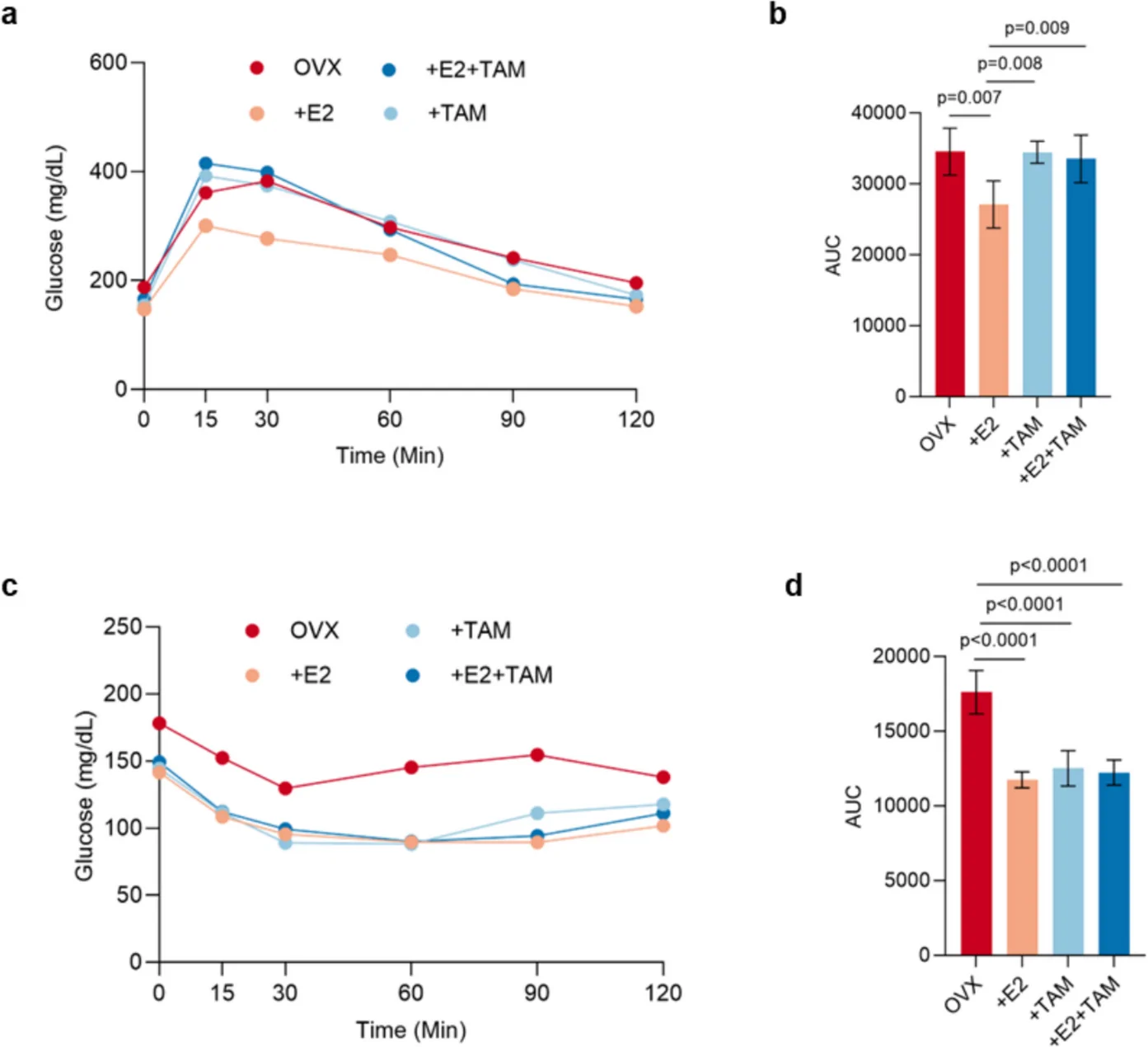
Tamoxifen impairs glucose tolerance but preserves insulin sensitivity following ovariectomy. **a** Intraperitoneal glucose tolerance test (IPGTT) showing changes in blood glucose levels (mg/dL) over 120 min post-glucose injection. **b** Area under the curve (AUC) for IPGTT, representing total blood glucose levels over time. **c** Insulin tolerance test (ITT) showing changes in blood glucose levels (mg/dL) over 120 min post-insulin injection. **d** Area under the curve (AUC) for ITT, indicating insulin sensitivity. One-way ANOVA, Sidak’s multiple comparison test; data are presented as mean ± SEM; each point and bar represent group average. OVX, *n* = 9; + E2, *n* = 8; + TAM, *n* = 9; + E2 + TAM, *n* = 9

Glucose tolerance was impaired in the OVX, + TAM, and + E2 + TAM treatment groups compared to the + E2-treated mice. At the 120-min mark of the glucose tolerance test, the OVX mice had blood glucose concentrations in the pre-diabetic range (196 ± 44 mg/dL) [49], while the + E2 mice maintained glucose concentrations within the normal range (152 ± 13.8 mg/dL) (Fig. 4a–b). OVX mice also reduced insulin sensitivity, whereas the + E2, + TAM, and + E2 + TAM groups remained insulin-sensitive (Fig. 4c–d).

**Fig. 5:**
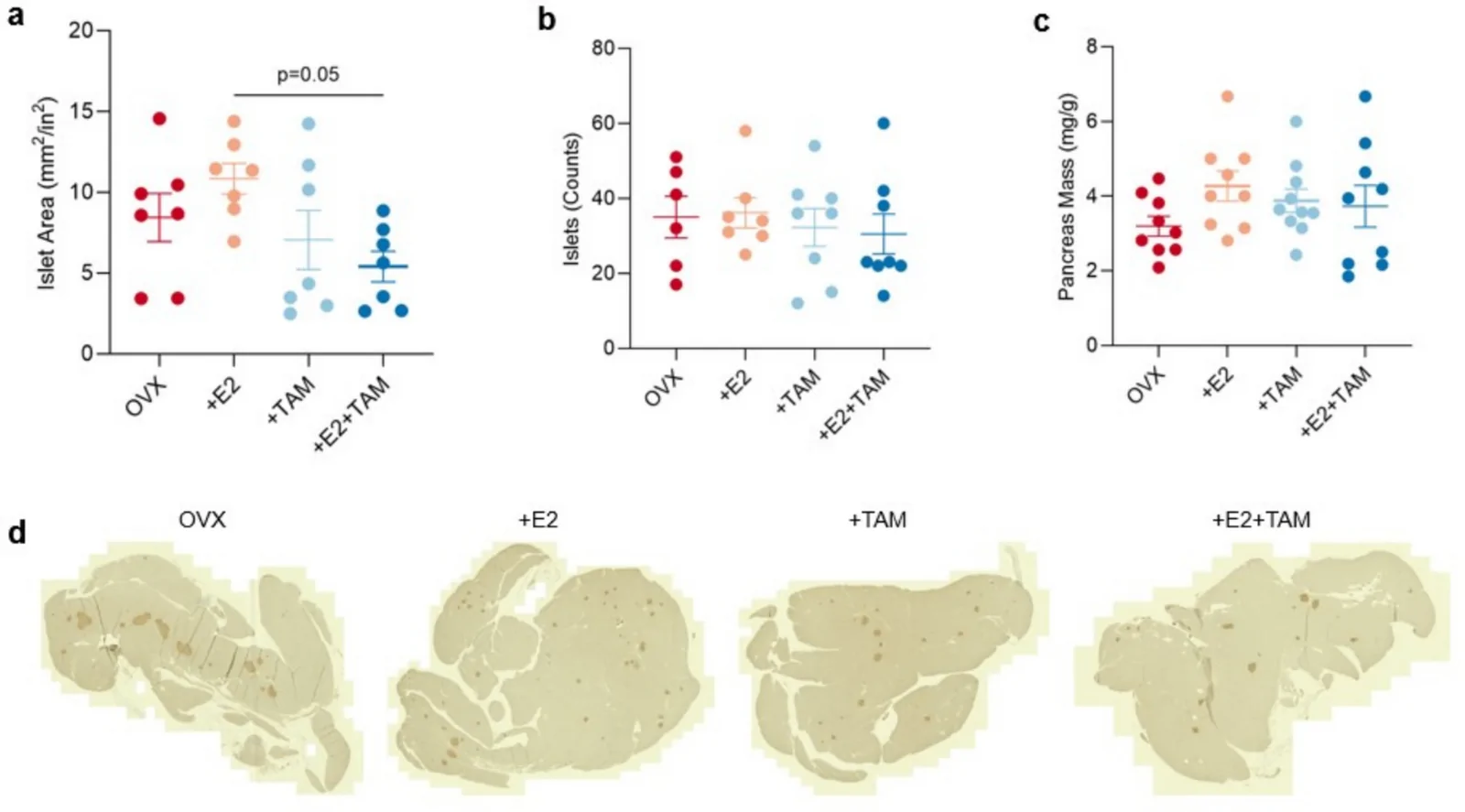
Tamoxifen impairs insulin-secreting pancreatic islets. **a** Total insulin-positive islet area normalized to total pancreatic area (mm^2^/in^2^), representing β-cell insulin secretory capacity; **b** total number of insulin-positive islets per pancreas section; **c** pancreatic mass normalized to body weight (mg/g); and **d** representative images of insulin + islet (dark brown) pancreatic tissue (tan) IHC staining at × 20 magnification across treatment groups: OVX (left), + E2 (center left), + TAM (center right), and + E2 + TAM (right). One-way ANOVA, Sidak’s multiple comparison test; data are presented as mean ± SEM. OVX, *n* = 9; + E2, *n* = 8; + TAM, *n* = 9; + E2 + TAM, *n* = 9

Histological analysis of insulin-secreting pancreatic islets revealed that the + E2 mice had twice the pancreatic islet area compared to the + E2 + TAM group, suggesting impaired insulin secretion in the latter that supports the observed glucose intolerance (Fig. 5a). The number of islets and pancreatic mass were unaffected across all groups (Fig. 5b–c).

### Tamoxifen alleviates OVX-induced proinflammatory milieu

**Fig. 6:**
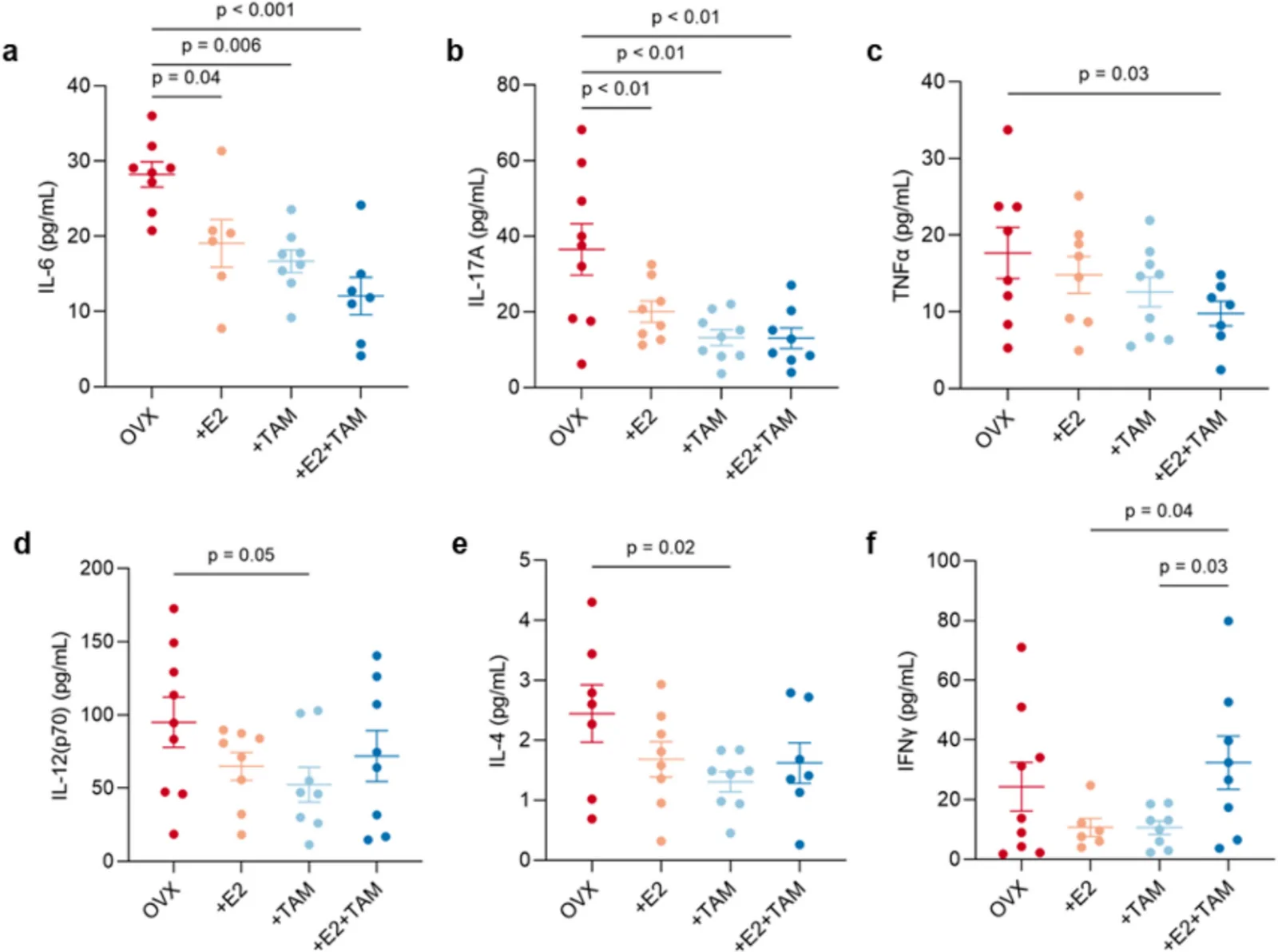
Tamoxifen ameliorates OVX-induced systemic inflammation. To assess treatment effects, serum concentrations of key cytokines were measured in each group at study endpoint: **a** interleukin-6 (IL-6), **b** interleukin-17A (IL-17A), **c** tumor necrosis factor alpha (TNF-α), **d** interleukin-12 (IL-12p70), **e** interleukin-4 (IL-4), and **f** interferon gamma (IFN-γ). Each dot represents an individual animal. Data are presented as pg/mL. One-way ANOVA, Fisher’s LSD test; data are presented as mean ± SEM. OVX, *n* = 9; + E2, *n* = 8; + TAM, *n* = 8; + E2 + TAM, *n* = 7–9

Adipose tissue accretion is a key driver of inflammation, which is further exacerbated by OVX. We examined the effects of TAM and E2 + TAM on OVX-induced systemic inflammation. OVX mice exhibited elevated levels of pro-inflammatory cytokines, including IL-6 and IL-17A, which were significantly reduced in the + E2, + TAM, and + E2 + TAM groups (Fig. 6a–b). TNF-α levels were also decreased across treatment groups compared to OVX mice, with statistical significance observed only in the + E2 + TAM group. TNF-α levels were 80.6% higher in OVX mice (Fig. 6c). IL-12(p70) levels trended higher in OVX mice compared to all treatment groups, with statistical significance observed only in comparison to + TAM, where OVX exhibited a 1.81-fold increase (Fig. 6d). Interestingly, IL-4, a cytokine with both pro- and anti-inflammatory roles, was significantly reduced only in the + TAM-treated mice (Fig. 6e). Conversely, IFN-γ was three times higher in the + E2 + TAM group compared to the + E2 and + TAM groups (Fig. 6f). These findings suggest that TAM, as part of a tissue-selective estrogen complex, effectively mitigates OVX-induced inflammation.

### Tamoxifen modifies adipose tissue B cell molecular phenotype

**Fig. 7:**
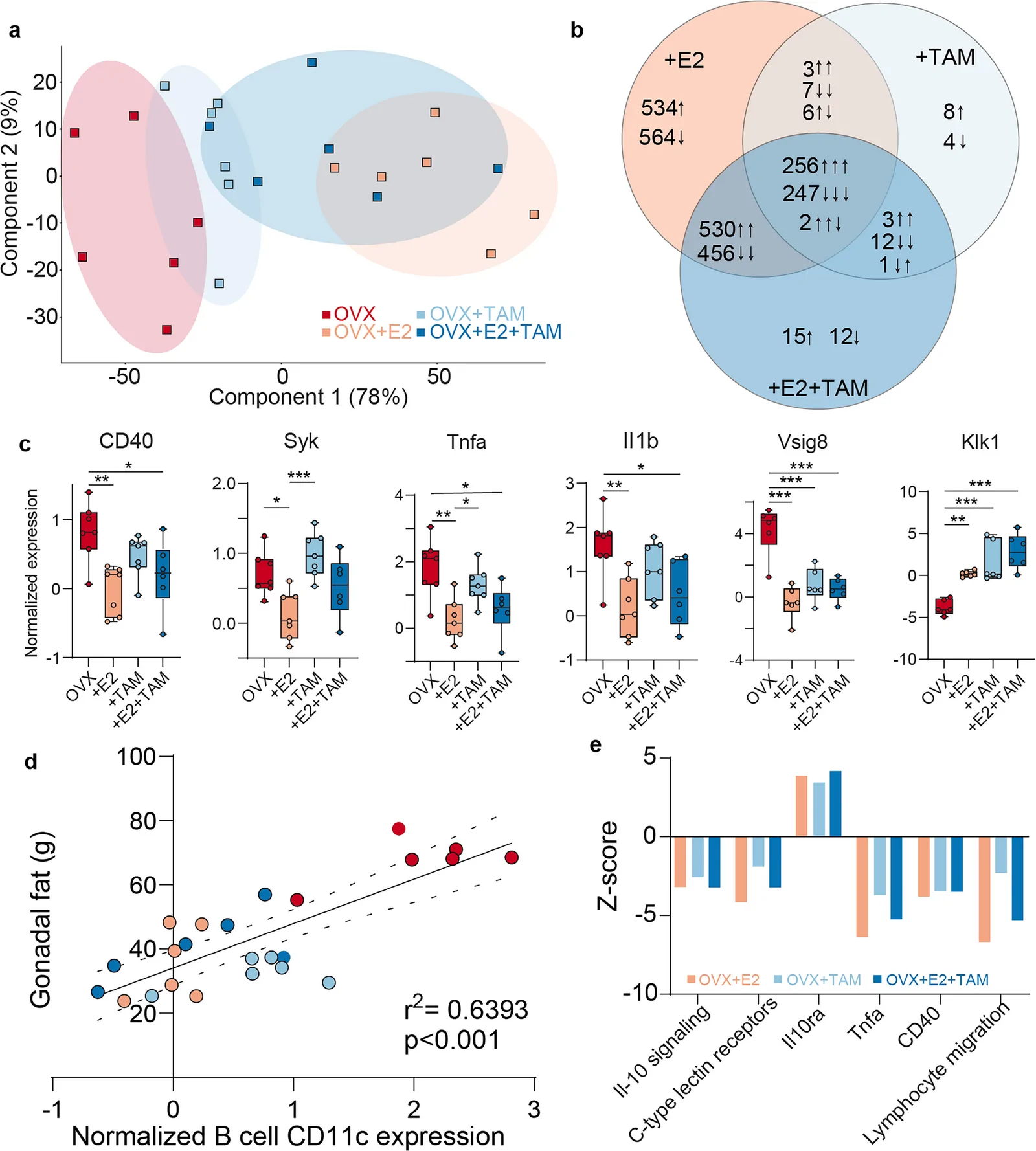
Tamoxifen alters the transcriptional profile of adipose tissue B cells. Transcriptomic analysis of B cells isolated from visceral adipose tissue from each study group: **a** principal component analysis, **b** DEGs Venn diagram by group, **c** gene expression of individual targets, **d** correlation analysis between gonadal fat and Cd11c gene expression, and e ingenuity pathway analysis by group compared to OVX; *n* = 6 per group. One-way ANOVA with Benjamini Hochberg multiple testing correction, Student Newman Keuls pairwise post hoc test; *p < 0.05; **p < 0.01; ***p < 0.001

We investigated the molecular phenotype of B cells isolated from perigonadal adipose tissue, which is associated with metabolic dysfunction and a surrogate for human visceral adipose tissue, using RNA sequencing (*n* = 6–7 group). Transcriptomic analysis revealed that B cell gene expression profiles clustered along a continuum from OVX to + TAM to + E2 + TAM and + E2, indicating similar effects of E2 and TAM across treatments, albeit with varying magnitudes (Fig. 7a). Differentially expressed genes (DEGs) showed commonalities in the genes’ changed in expression and the direction of change (up/down regulated) across treatment groups, suggesting that both E2 and TAM modulate B cell function in a similar manner, although to different extents (Fig. 7b). We next examined selected genes from the transcriptomic analysis with relevance to B cell activation, cytokine production, and immune regulation (Fig. 7c). CD40, a costimulatory receptor expressed by activated B cells, is essential for germinal center formation, plasma cell and memory B cell development, and cytokine production in coordination with B cell receptor signaling [50, 51]. Elevated CD40 signaling has been linked to increased pro-inflammatory cytokine production [52] and autoimmune pathology [53]. In our study, CD40 expression in visceral AT B cells was reduced in the + E2 and + E2 + TAM groups but remained elevated in + TAM alone. A similar trend was observed for Tnfα and Il1β (Fig. 7c), with reductions in all treatment groups but a less pronounced effect in + TAM, suggesting that estradiol more effectively attenuates the pro-inflammatory B cell molecular phenotype. Syk, a key mediator of BCR signaling, was decreased by + E2, but this effect was lost with TAM co-treatment, suggesting potential interference with E2-mediated suppression of B cell activation. Vsig8, a co-inhibitory ligand for the T cell checkpoint receptor VISTA (V-domain Ig suppressor of T cell Activation) that modulates adaptive immune responses [54, 55], was significantly reduced in all treatment groups relative to OVX, indicating possible dampening of B cell–T cell interactions and modifying the adaptive immune response in adipose tissue. Conversely, Klk1, a serine protease regulated by estrogen and involved in vascular remodeling, angiogenesis, and tissue homeostasis [56], was significantly increased in the + E2, + TAM, and + E2 + TAM groups, suggesting a potential role in AT biology.

Notably, expression of CD11c^+^, a defining marker of age-associated B cells (ABCs), was positively correlated with gonadal fat mass (Fig. 7d). ABCs, characterized by high CD11c^+^CD27^lo^CD21^lo^CD23^lo^T-bet^+^ (humans) and CD11c ^+^ T-bet ^+^ (mouse) [57], expand in chronic immune activation states including autoimmunity, infection, and obesity. In mouse models of diet-induced obesity, T-bet ^+^ ABCs promote metabolic disease and accumulate in AT. The positive correlation observed here suggests that CD11c^+^ B cells may infiltrate AT during weight gain, contributing to low-grade inflammation. In addition, examination of regulator effects by Ingenuity Pathway Analysis demonstrated similar downstream regulation patterns for different inflammatory modulators across all treatments and indicative of suppressed inflammation (Fig. 7e).

### Tamoxifen and ovariectomy modulate circulating immunoglobulin G subclasses concentrations

**Fig. 8:**
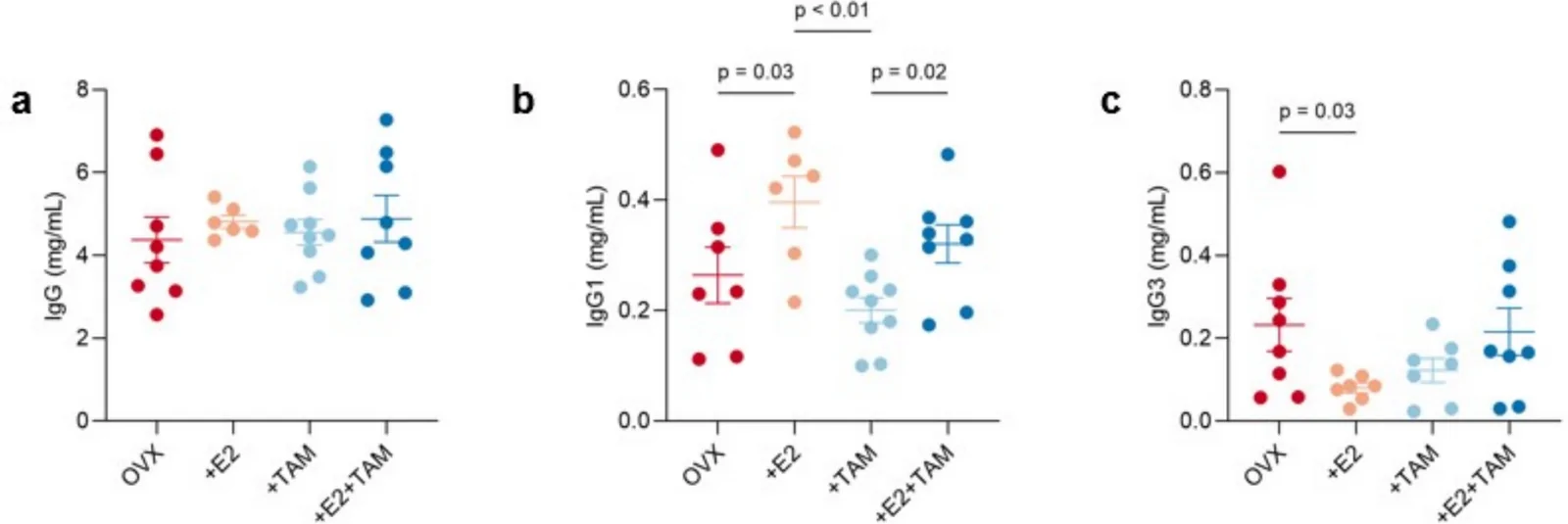
Tamoxifen and ovariectomy modifies circulating immunoglobulin G subclasses: **a** total plasma IgG levels presented in mg/mL, **b** plasma IgG1 levels in mg/mL, and **c** plasma IgG3 levels in mg/mL; one-way ANOVA, Fisher’s LSD test. Data are presented as mean ± SEM. OVX, *n* = 8; + E2, *n* = 7; + TAM, *n* = 9; + E2 + TAM, *n* = 8

We quantified plasma IgG, IgG1, and IgG3 to assess how OVX and ER modulation influence systemic antibody levels. While we saw no difference in total circulating plasma IgG levels (Fig. 8a), OVX mice exhibited significantly reduced IgG1 concentrations compared to the + E2-treated mice. TAM treatment similarly suppressed IgG1, whereas E2 replacement, either alone or combined with TAM, restored IgG1 levels (Fig. 8b). In contrast, OVX mice had elevated plasma IgG3 levels compared to the + E2-treated mice (Fig. 8c). No further statistically significant differences were seen between groups.

## Discussion

This study investigated the effects of estrogen deprivation and estrogen receptor modulation by low-dose tamoxifen (TAM) on body composition, adiposity, metabolic function, and B cell biology. Specifically, we examined whether TAM could mitigate ovariectomy-induced metabolic dysfunction in adult female mice by modulating E2–ER signaling. We also assessed how estrogen deprivation and TAM influence the molecular phenotype of B cells in visceral adipose tissue. OVX led to signs of metabolic dysfunction, including weight gain, fat accumulation with enlarged adipocytes, increased ectopic lipid deposition, systemic inflammation, glucose intolerance, and decreased insulin sensitivity. However, treatment with + TAM alone or combined with + E2 improved several of these outcomes in a manner similar to E2 replacement. Despite these improvements, glucose intolerance persisted, although insulin sensitivity in tissues remained intact in the + TAM and + E2 + TAM groups. We then analyzed the impact of TAM and TAM + E2 on the gene expression profile of visceral AT B cells. The patterns of differentially expressed genes revealed overlaps in both gene identity and direction of change, indicating that TAM and E2 elicit similar responses in B cells. Overall, our findings suggest that TAM mimics the beneficial effects of E2 in regulating body composition, inflammation, and adaptive immune B cell function.

One of the hallmark physiological changes associated with menopause is a shift in body composition, particularly an increase in adiposity and central fat accumulation [6]. These changes are closely linked to the decline in circulating 17β-estradiol (E2), which alters energy balance by reducing energy expenditure, increasing caloric intake, and redistributing fat deposition toward the abdominal region [58, 59]. As a result, nearly 50% of postmenopausal women are overweight, with approximately 25% classified as obese [60]. The loss of E2 has been implicated in multiple mechanisms contributing to this increase in adiposity, including impaired appetite regulation and reduced metabolic rate [61, 62]. Both clinical and preclinical studies have established a key role for E2 in maintaining energy homeostasis and body composition, and its depletion is increasingly recognized as a driver of age-related metabolic dysfunction [8, 63]. Our findings support this model, as OVX mice gained significantly more fat mass and overall body weight compared to all other treatment groups. This observation is consistent with prior reports demonstrating that E2 depletion promotes fat accumulation, while E2 replacement mitigates this effect. Interestingly, we found that TAM treatment prevented weight and fat gain in OVX mice, aligning with studies suggesting that TAM can act as an E2 agonist in certain metabolic tissues [64]. However, the literature on the metabolic effects of SERMs shows mixed results. While some studies report TAM-induced fat loss [65], others have described weight gain [66], often confounded by concurrent cancer treatments. Notably, a comprehensive study that sought to disentangle the effects of TAM from concurrent breast cancer treatments reported that TAM reduced waist circumference, weight, and BMI when administered to healthy premenopausal women, suggesting context-dependent metabolic effects [67].

Postmenopausal women have a significantly higher prevalence of metabolic syndrome (MetS) compared to their premenopausal counterparts, with estimates ranging from 31 to 55% versus 20 to 25% of the adult population [68, 69]. MetS is defined by a constellation of metabolic abnormalities which include central obesity, dyslipidemia, hypertension, insulin resistance (IR), and chronic inflammation that collectively increase the risk of cardiovascular disease and T2DM. Estrogen deficiency is a key factor contributing to the increased incidence of MetS in postmenopausal women [70].

Aging is associated with a progressive decline in glucose tolerance [71], and menopause further exacerbates this through impaired insulin signaling and regulation [72]. Consequently, T2DM is highly prevalent during the postmenopausal period [73]. Dysregulation of glucose metabolism may arise from several mechanisms, including reduced insulin secretion from pancreatic islets [74], beta-cell dysfunction [75], and decreased peripheral insulin sensitivity [76]. HRT with estrogen has been shown to delay the onset of T2DM by improving beta-cell insulin secretion, glucose effectiveness, and insulin sensitivity [77]. A meta-analysis of 17 trials involving over 29, 000 postmenopausal women confirmed that estrogen-based HRT significantly reduces IR in healthy individuals [78]. In murine models, E2 has been shown to suppress hepatic gluconeogenesis and enhance insulin sensitivity through a liver-specific FOXO1-dependent mechanism [79]. It also demonstrated that loss of E2 signaling compromises metabolic homeostasis in both sexes, resulting in elevated fasting glucose and reduced insulin sensitivity—paralleling our findings in OVX versus E2-treated mice. Further mechanistic insight revealed that hepatic estrogen signaling plays a central role in regulating glucose metabolism, as deletion of hepatic ERα impaired glucose tolerance and insulin sensitivity in both male and female mice [80]. These effects were attributed to estrogen-mediated stabilization of IRS1 protein, emphasizing the importance of hepatic ERα signaling in preserving metabolic balance.

While the metabolic effects of E2 are well characterized, reports on the effects of SERMs are inconsistent. Some studies have shown that TAM improves glucose and insulin tolerance as well as hepatic insulin signaling [81, 82], while others report sex-specific effects, with metabolic improvements in males and impairments in females [83]. Our findings demonstrate that E2 replacement recovers OVX-induced glucose intolerance and maintains peripheral insulin sensitivity under HF/HS dietary intake. OVX mice showed both glucose intolerance and decreased insulin sensitivity. Interestingly, + TAM and + E2 + TAM groups, which exhibited impaired glucose tolerance, maintained insulin sensitivity and had significantly less insulin-secreting islet area compared to the + E2 mice. These findings suggest that TAM may impair insulin secretion capacity, contributing to worsened glucose metabolism independent of peripheral insulin sensitivity.

Ectopic lipid accumulation in the liver is a key contributor to the pathogenesis of metabolic syndrome (MetS) and is closely regulated by estrogen signaling [84, 85]. Postmenopausal women face a significantly heightened risk of developing metabolic dysfunction-associated fatty liver disease (MAFLD), with odds approximately 2.4 times higher than those of premenopausal women [86]. Estrogen plays a protective role in hepatic lipid metabolism by suppressing de novo lipogenesis and enhancing lipid clearance [87]. Supporting this, clinical data show that estrogen-based HRT is associated with a 13.5% reduction in the prevalence of MAFLD [88].

In contrast, TAM has been reported to exacerbate hepatic lipid accumulation in both preclinical and clinical contexts. In rodent models, TAM combined with high-fat feeding promotes the development of metabolic dysfunction-associated steatohepatitis (MASH) [89]. Similarly, in human studies, patients receiving TAM for breast cancer treatment exhibit an increased incidence of fatty liver disease [90]. However, murine models suggest a dose-dependent relationship in which high-dose TAM (100 mg/kg/day for 8 weeks) attenuates high-fat diet-induced hepatic steatosis while maintaining hepatocyte function and protecting against lipotoxicity [82]. In our study, both the OVX and + TAM groups exhibited significantly elevated hepatic lipid deposition compared to the + E2 and + E2 + TAM-treated mice. Notably, despite reduced adiposity in the + TAM group, hepatic lipid levels remained similar to OVX-only animals, suggesting a dissociation between systemic fat mass and hepatic lipid handling under low-dose TAM treatment. These findings highlight the tissue- and dose-selective effects of TAM and underscore the complexity of its systemic metabolic profile. Liver lipid accumulation is a known contributor to IR, as it impairs hepatic insulin signaling, increases glucose production, and decreases glucose utilization, thereby elevating the risk for developing T2DM [84]. ERα signaling in the liver plays a protective role in glucose homeostasis and has been shown to improve glucose tolerance and insulin sensitivity in murine models [69, 70]. In our study, glucose intolerance was observed in the OVX, + TAM, and + E2 + TAM groups; however, increased hepatic lipid accumulation was restricted to the OVX and + TAM groups. These findings suggest that while TAM impairs glucose tolerance, E2 may confer protection against hepatic steatosis in the + E2 + TAM group, possibly through hepatic ER signaling.

Postmenopausal women experience marked changes in immune function, driven by both aging and the decline in circulating estradiol (E2) levels [16, 17]. Menopause is associated with reduced B and T cell numbers, diminished immune cell activity, and a shift toward a pro-inflammatory state [17]. E2 plays a regulatory role in both the innate and adaptive arms of the immune system [13], and reciprocal interactions between the endocrine and immune systems further complicate this relationship. Immune cells express estrogen receptors (ERs) [91], and cytokine receptors are present on estrogen-producing cells [92], suggesting bidirectional signaling between different cell types. ER modulation has been shown to impact immune cell development, phenotype, and function [13, 93], and the loss of E2 during menopause contributes to immune dysregulation by shifting immune cells into a pro-inflammatory state [94]. Immune dysfunction in menopause is further compounded by the effects of obesity. AT is recognized as an immunologically active organ. Obesity drives the infiltration of B cells, T cells, and macrophages into AT, promoting a chronic low-grade inflammatory microenvironment [18–20]. B cells have emerged as key contributors to metabolic dysfunction, acting through antigen presentation, secretion of pro-inflammatory cytokines, and production of pathogenic antibodies, all of which contribute to insulin resistance and the progression to T2DM [95]. It has been shown that B cell deficiency improves metabolic outcomes in mice; B cell-deficient (B6 muMt^−^) mice exhibited lower fasting glucose and enhanced insulin sensitivity, while the adoptive transfer of B cells from diet-induced obese wild-type mice restored metabolic dysfunction [96]. These findings underscore the immunometabolic role of B cells and highlight their responsiveness to hormonal regulation. E2 has been shown to influence B cell biology by regulating their differentiation, activation, and survival [97]. Given this, HRT has been proposed as a strategy to reverse immune aging in postmenopausal women, potentially restoring B cell numbers and enhancing T cell responsiveness [34]. However, the immunological effects of SERMs and TSECs remain poorly understood. In this context, we examined the impact of estrogen deprivation, E2 replacement, and ER modulation by TAM on visceral AT B cell molecular phenotype. Transcriptomic analysis revealed that AT-B cells in OVX mice exhibited increased activity and immune signaling compared to E2-treated animals, supporting the notion of heightened immune activation in the absence of estrogen. These immunological shifts were accompanied by systemic changes in the inflammatory environment. Serum cytokine analysis revealed elevated levels of IL-6, TNFα, IL-12(p70), and IL-17A in OVX mice, consistent with the development of a pro-inflammatory milieu. In contrast, + E2 and + TAM treatments reduced systemic inflammatory markers, suggesting that modulation of ER signaling can influence both local immune cell phenotype and systemic inflammatory status.

We evaluated the humoral component of systemic immunity by quantifying circulating total IgG, IgG1, and IgG3, the predominant immunoglobulin isotype and subclasses responsible for broad pathogen neutralization and opsonization [98]. IgG1, which constitutes approximately 60–65% of total IgG [99], mediates the majority of IgG-dependent responses to protein antigens. OVX significantly reduced circulating IgG1 concentrations, and a similar suppression was observed with + TAM treatment, indicating that estrogen deprivation and ER modulation impair IgG1 homeostasis. In contrast, E2 replacement, either alone or combined with TAM, restored IgG1 levels, highlighting the dependence of IgG1 regulation on estrogen signaling. Clinically, IgG1 deficiency is associated with recurrent infections [100] and contributes to hypogammaglobulinemia, which compromises antiviral and antibacterial defense [101]. Reduced total IgG may arise from impaired B cell development, defective class-switch recombination, or increased immunoglobulin loss [102, 103]. Together, these findings suggest that estrogen loss and TAM-mediated ER modulation diminish humoral immune capacity, whereas E2 replacement restores immunoglobulin homeostasis and may help preserve antibody-mediated immunity during menopause.

We also observed elevated circulating IgG3 concentrations in OVX mice compared to those receiving E2 replacement. IgG3 is distinguished from other IgG subclasses by its unusually long hinge region, which confers increased molecular flexibility, potent Fc-mediated effector functions, and additional O-glycosylation sites that enhance antigen engagement [104]. Although it represents a relatively small fraction of total immunoglobulins, IgG3 plays a disproportionately important role in early immune defense against bacterial, viral, and parasitic pathogens. However, sustained elevations in IgG3 are associated with heightened inflammatory responses and have been linked to increased disease severity in infections such as tuberculosis and dengue virus [104, 105]. Excessive IgG3 signaling has also been shown to negatively regulate memory B cell formation and impair B cell receptor responsiveness [101, 106]. Moreover, elevated IgG3 can serve as a biomarker of autoimmune activity, including in systemic lupus erythematosus, where increased levels reflect ongoing systemic inflammation [107].

Together, the suppression of IgG1 and concomitant elevation of IgG3 in OVX mice indicate a shift toward a sustained pro-inflammatory humoral immune state, consistent with heightened susceptibility to infection and inflammatory disease. E2 replacement shifts both IgG1 and IgG3 levels toward maintaining balanced humoral immunity. These findings align with clinical observations that the menopausal transition is associated with increased systemic inflammation and a greater risk for autoimmune and infectious disease exacerbation.

Future studies should focus on the effects of low-dose tamoxifen on energy metabolism and adipocyte biology in aged female and male mice. As low-dose tamoxifen (5 mg daily for 3 years) has been shown to significantly reduce the risk of recurrence in women with noninvasive breast cancer without long-term adverse events, it is critical to evaluate short and long-term systemic inflammatory and immune status in breast cancer survivors.

## Conclusions

Despite menopause being a universal stage in aging with health implications, the full extent of its effects on whole-body metabolism and immunity remains underexplored. Similarly, the systemic and immunologic impacts of estrogen receptor modulation by SERMs and TSECs are not well understood. In this study, we examined how estrogen loss and ER modulation influence body composition, metabolic function, and immune responses in a mouse model of menopause. Our findings reveal that both 17β-estradiol replacement and the TSEC (E2 + TAM) effectively mitigate key menopause-associated risks, including obesity, insulin resistance, and ectopic liver lipid deposition. However, TAM alone worsened metabolic outcomes, particularly glucose intolerance. Additionally, menopause-induced immune changes, such as a shift toward a pro-inflammatory state of B cells, were partially reversed by E2 replacement, alone and in combination with TAM. This research contributes to a more comprehensive understanding of how menopause and its treatments influence metabolism and immunity, opening avenues for further exploration of therapeutic strategies.

## Supplementary information

Below is the link to the electronic supplementary material.

Supplementary file1 (DOCX 175 KB)

## Data Availability

The authors confirm that all data supporting the findings of this study are included within the article and its supplementary materials and available from the corresponding author upon request.
