# Supplementary material for: Low-dose tamoxifen ameliorates ovariectomy-induced metabolic and immune dysfunction
Source: GeroScience. 2026 Feb 11;48(6):8413–34. doi: 10.1007/s11357-025-02086-7 (PMC13638879; doi:10.1007/s11357-025-02086-7)
Supplement: Supplementary file 1 [file 11357_2025_2086_MOESM1_ESM.docx]

**Supplemental Fig. 1 Confirmation of estrogen replacement** **(a)** Uterine mass, normalized to body weight, served as a bioassay for estrogenic activity. **(b)** Circulating 17β-estradiol levels measured at study endpoint confirmed successful hormone delivery, with elevated E2 levels in the +E2 and +E2+TAM groups. Data are presented as pg/mL. One-way ANOVA, Sidak’s multiple comparison test. Data are presented as mean ± SEM. OVX n=9, +E2 n=8, +TAM n=9, +E2+TAM n=9
